# Empathic concern drives costly altruism

**DOI:** 10.1016/j.neuroimage.2014.10.043

**Published:** 2015-01-15

**Authors:** Oriel FeldmanHall, Tim Dalgleish, Davy Evans, Dean Mobbs

**Affiliations:** a Medical Research Council, Cognition and Brain Sciences Unit, 15 Chaucer Road, Cambridge CB2 7EF, UK; b Columbia University, Department of Psychology, 370 Schermerhorn Hall 1190 Amsterdam Ave., New York, NY 10027, USA

**Keywords:** Moral, Caudate, Subgenual ACC, VTA, Empathy, Altruism, Distress

## Abstract

Why do we self-sacrifice to help others in distress? Two competing theories have emerged, one suggesting that prosocial behavior is primarily motivated by feelings of empathic other-oriented concern, the other that we help mainly because we are egoistically focused on reducing our own discomfort. Here we explore the relationship between costly altruism and these two sub-processes of empathy, specifically drawing on the caregiving model to test the theory that trait empathic concern (e.g. general tendency to have sympathy for another) and trait personal distress (e.g. predisposition to experiencing aversive arousal states) may differentially drive altruistic behavior. We find that trait empathic concern – and not trait personal distress – motivates costly altruism, and this relationship is supported by activity in the ventral tegmental area, caudate and subgenual anterior cingulate, key regions for promoting social attachment and caregiving. Together, this data helps identify the behavioral and neural mechanisms motivating costly altruism, while demonstrating that individual differences in empathic concern-related brain responses can predict real prosocial choice.

## Highlights

• Trait empathic concern predicts altruistic action.
• State distress, but not trait distress, predicts altruistic action.
• A network comprised of VTA, caudate and sgACC support other-oriented prosocial decisions.
• Neural evidence supporting caregiving model: social reward and attachment guides altruism.

## Introduction

On a daily basis we are inundated with powerful media images of child famine, domestic violence, and natural disasters. Beyond informing society, one aim of such ‘shock tactic’ images is to evoke feelings of distress in the observer with the desire of increasing charitable donations. This is supported by a theory suggesting that altruistic behavior – helping others at a cost to the self (de Waal, 2008) – is a function of the desire to minimize one's own discomfort when observing others in pain (Cialdini et al., 1987). Alternatively, the empathy–altruism hypothesis proposes that ‘other-oriented’ empathic emotions, such as sympathy, are better predictors of altruistic behavior (Batson et al., 1983). Combining brain imaging and a paradigm that requires subjects to take action to reduce a stranger's pain at a financial cost to oneself, we sought to explore the underlying psychological and neural mechanisms that motivate altruistic action when self-interest is at stake: are we primarily driven to help at a cost to ourselves because we experience other oriented feelings of concern, or mainly to alleviate our own discomfort when seeing another in pain?

Empathy – the capacity to have feelings that reflect the emotional dynamics of another's situation (Hoffman, 2000) – is a multidimensional psychological concept (Zaki and Ochsner, 2012) comprised of distinct, yet related, cognitive and affective processes (Davis, 1983; Batson et al., 1987; Preston, 2002; Penner et al., 2005). In other words, although these empathy components are thought to share a common affective base (Preston, 2002), personal distress (aversive arousal states), empathic concern (most closely associated with sympathy), and perspective-taking (a more cognitive process), can each lead to different emotional (Eisenberg et al., 1994) and behavioral patterns (Batson et al., 1987). For example, personal distress is more often associated with self-directed behavior (escaping a distressing situation to terminate one's own distress), while empathic concern involves the orientation towards another's needs (relieving or attenuating the distress of another) (Eisenberg et al., 1994). However, research linking these empathic processes to altruistic behavior has revealed mixed findings: some studies report that empathic concern motivates prosocial behavior while personal distress is more predictive of avoidant behavioral patterns (Batson et al., 1987, 1988; Eisenberg et al., 1994; Eisenberg, 2000). Conversely, other work suggests that vicarious distress is a necessary antecedent of prosocial choice (Cialdini et al., 1987, 1997), and more recent research illustrate that aversive arousal states (i.e. personal distress) lead to costly helping (Hein et al., 2011).

Evidence of such vicariously experienced distress has also been demonstrated at the neural level. A decade of brain imaging studies has led to a replicable neural circuitry that is activated in a variety of empathic situations (Singer et al., 2004; Lamm et al., 2011), and includes the bilateral anterior insular (AI) and dorsal anterior cingulate cortices (dACC) — key regions in the brain's response to physical pain (Singer et al., 2004; Lamm et al., 2007; Akitsuki and Decety, 2009). This reliable ‘empathy for pain’ network illustrates that the experiences of self and other pain are intimately associated — if you suffer, I too suffer (Decety et al., 2008). Evidence of such vicarious distress (i.e. personal distress) is often taken as an indication that subjects are experiencing empathic concern (Singer et al., 2004, 2006; Singer and Frith, 2005). Theorists, however, have posited that the existence of a neural overlap found during self-other pain paradigms should not be taken to ubiquitously signify the presence of the more emotional component of empathic concern (Preston, 2002). This confusion over the way related empathic phenomena map onto neural processes has made it difficult to understand if the empathy for pain network activates because individuals are experiencing self-focused personal distress or other-oriented empathic concern (Bernhardt and Singer, 2012).

A further unresolved issue relates to the neural mechanisms supporting these various empathy components and the link to costly altruism (de Vignemont and Singer, 2006). Since many empathy paradigms traditionally measure brain activation while subjects passively view others in pain (Singer et al., 2004, 2006; Lamm et al., 2007, 2008; Xu et al., 2009; Decety et al., 2010), less is known about the neural mechanisms underpinning helping behavior when self-interest is at stake (Hein et al., 2010). Extending prior work investigating pain and its relationship to empathy (Lamm et al., 2007), we tested these questions by combining functional magnetic resonance imaging (fMRI) and a highly distressing social interaction paradigm (the Pain vs. Gain [PvG] task) where subjects are required to decide between financial self-benefit and ensuring the physical welfare of another, a trade-off that is characteristic of many decisions we make in everyday life (FeldmanHall et al., 2012b). During the task, subjects are explicitly presented with the choice to help targets in relatively high distress — a key precondition for altruistic responding (Preston, 2013). We reasoned that such a complex and costly social interaction would likely provoke nuanced empathic processes (Zaki and Ochsner, 2012), allowing us to explore the brain–behavior relationship between individuals' decisions to act prosocially or selfishly, and the underlying affective mechanisms motivating such preferential action.

That both Personal Distress and Empathic Concern have previously been associated with prosocial responding mandated that our first goal to identify if these empathic states differentially motivate altruistic action when there is a cost to the self. We also entertained the possibility that altruistic action may require some combination of these empathy components. To test these behavioral hypotheses, we employed the PvG task in conjunction with a well-established trait measure of Personal Distress and Empathic Concern (Davis, 1983) and explored whether these psychological states differentially correlate with altruistic action.

Based on the neural mechanisms instantiated in the caregiving model (Preston and de Waal, 2011; Preston and Hofelich, 2012; Preston, 2013), we hypothesized that if we found evidence that empathic sub-processes rely on separate neural circuitry, it would support the notion that these two psychological states differentially motivate altruistic action. If this were indeed the case, the care-giving model (Preston, 2013) proposes that a pattern of avoidant (i.e. selfish or no helping) behavior would likely engage the dACC, amygdala, and periaqueductal gray (PAG) — regions key for processing conflict and which have been shown to correspond with negative emotions and avoidant maternal expressions (Numan, 2006). In contrast, a candidate network for processing behavioral approach patterns, are the dopamine rich mesolimbic regions (Preston, 2013), which include the ventral tegmental area (VTA), caudate, and Nucleus Accumbens (NAcc) — areas that are tied to altruistic giving (Kosfeld et al., 2005; Moll et al., 2006; Harbaugh et al., 2007) and the ‘warm glow’ feeling that is experienced when helping others (Andreoni, 1990). In addition, the subgenual cingulate (sgACC) is an area associated with regulating emotional responses, making it a prime region to directly mediate helping behavior (Preston and de Waal, 2011). However, if aversive arousal states and empathic concern are both necessary antecedents to motivate altruistic action, then it is possible that we would find neural activation in more cortical modulatory systems (e.g. dorsolateral prefrontal cortex) which are heavily recruited when multiple responses (aversive distress and empathic concern) must be integrated into a cost benefit analysis to generate subsequent behavior (Preston, 2013).

## Materials and methods

### Subjects

19 subjects took part in this study. Two subjects were excluded from analyses due to expressing doubts about the veracity of the PvG task on a post-scan questionnaire and during debriefing. For the participants who completed the PvG task and who were included in analyses (N = 17, 6 males; mean age and SD 23.3 ± 3.1), there was no significant correlation between their ratings of the believability of the task and their behavioral performance (shock delivered/Money Kept), Pearson's correlation r = − .21, p = 0.42, 2-tailed.

### Pain vs. Gain (PvG) task

**Fig. 1:**
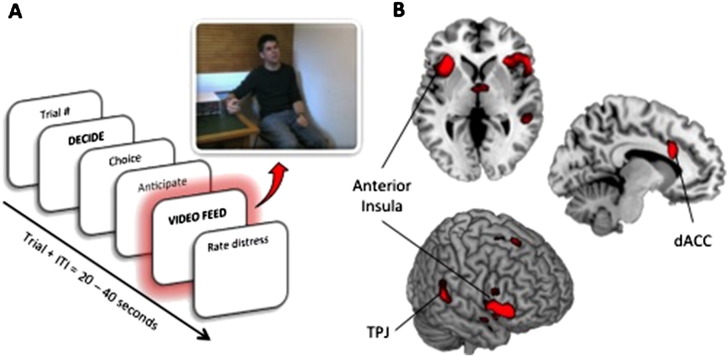
A. Trial sequence illustrating the analyzed epoch of the PvG task: Decide event and Video event. B. Observing the decision's outcome (Video event) reveals activation in the ‘empathy for pain’ network, including bilateral anterior insula (AI), and anterior cingulate (ACC) activity; temporoparietal junction (TPJ) activation was also found.

After being endowed with a personal bank account of £20 subjects (Deciders) were probed across 20 trials about their willingness to increase their financial gain (up to £200) at the expense of applying a series of harmful electric shocks to another subject (the Receiver — a confederate whom the Decider had met and interacted with). Deciders were also required to view via video feed the Receiver's facial and bodily reactions as the painful stimulations were administered (Video event [Fig. 1A]) before rating their own distress (a situational measure). Since a key prediction of the empathy–altruism hypothesis is that subjects are motivated to help, rather than escaping a distressing situation, we included an option for subjects to leave the experiment at any point, without compromising their hourly endowment.

The PvG task comprised a series of 8 screens per trial (Fig. 1A) across 20 trials. Each trial began with a screen displaying the running amount of the subject's bank total (£20 on Trial 1) and current trial number. Subjects then had up to 11 s to decide upon, and use a visual analogue scale (VAS) to select the amount of money they wanted to spend on that trial and thus the corresponding painful stimulation to be administered to the Receiver (Decide event). For example, a decision to keep £.80 on a given trial would result in a medium high shock being administered, whereas a decision to keep £.20 would result in the lowest shock level being administered. Decisions increased by £.20, starting with the choice to keep £1 (highest shock administered) to £0 (no shock administered). This 11 s phase was partitioned into the “Decide” and “Select” periods. The Decide screen was presented for a fixed 3 s during which subjects were asked to think about their decision, so that when the select screen appeared, subjects could move the cursor to make their selection any time within the next 8 s. This design was used in order to introduce a variable jitter within the trial sequence.

After making a selection, subjects saw a 3 s display of their choice before experiencing an 8 s anticipation phase — during which subjects were told their choice was being transmitted over the internal network to the other testing lab where the Receiver was connected to the electric stimulation generator. Following this anticipation period, subjects viewed a 4 s video of the stimulation being administered (Video event) to the Receiver, or no stimulation if they had opted to spend the full £1 permitted on a given trial. The Decider was able to see the entire face and body of the Receiver responding to the shock. The Video event was in fact pre-recorded footage of real shocks being administered to the Receiver, pre-rated by an independent group so as to be matched for shock level and corresponding pain intensity. The affective responses seen during the videos were graded such that increasing shock levels corresponded to distinctly distressed responses. Finally, subjects used a 13-point VAS to rate their situational distress levels on viewing the consequences of their decision, before viewing a 4 s inter-trial-interval (ITI). At the conclusion of the 20 trials, subjects were able to press a button to randomly multiply any remaining money between 1 and 10 times, thus giving a maximum possible financial gain of £200 from the task.

Subjects also completed a Non-Moral control task within the scanner which was used in the fMRI analysis as a baseline contrast against the PvG task. This task mimicked, both visually and structurally, the design of the PvG: in 8 screens per trial, across 20 trials, participants followed the same timings allotted in the PvG, only in this task they were asked to make a non-moral decision about which finger of the right hand the Receiver should move. This task matched the structural, temporal, and visual feedback of the PvG.

### Questionnaires

After the experimental session was finished, subjects answered a series of questions that asked them to indicate on 8-point analogue scales (ranging from 1 to 8): 1) whether they felt they were being watched during the experiment; 2) how much responsibility they (as Deciders), the experimenter, and the Receiver had for the shocks administered, 3) whether there was any doubt as to the veracity of the paradigm; 4) how much guilt subjects felt when making their decisions; 5) how likable they found the Receiver; and finally 6) their feelings towards the Receiver.

### Trait empathic concern and trait personal distress

We measured trait Empathic Concern and trait Personal Distress with the widely used Interpersonal Reactivity Index (IRI) (Davis, 1983). This questionnaire is divided into two dimensions, affective and cognitive, with each dimension containing two subscales. The affective dimension includes Empathic Concern – the tendency to experience feelings of sympathy or compassion for others, and Personal Distress – the tendency to experience distress or discomfort oneself in response to distress in others. We used these two subscales to measure dispositional, other-orientated concern and dispositional, personal ‘egoistic’ distress. Subjects' mean scores for the Empathic Concern subscale were slightly above average, however they were within the range of norm values: 26.6, SD ± 2.3; minimum score 23, maximum score 31. These scores were used as covariates of interest in second level fMRI analysis. Subjects' mean scores for Personal Distress were also slightly above average, however they were within the range of norm values: 18.8, SD ± 3.6; minimum score 13, maximum score 28. These scores were also used as covariates of interest in second level fMRI analysis. We found no intercorrelation between the Personal Distress and Empathic Concern subscales (r = − .03, p = .92) of the IRI, supporting the idea that these are separate empathy-related constructs (Batson et al., 1987).

#### Situational distress

We measured situational distress by asking subjects how they felt after making their decision for each trial on the PvG task and watching the outcome of their decision (i.e. the intensity of the Receiver's pain). Thus, we acquired situational distress ratings across all twenty trials. Subject mean scores: 8.2, SD ± 1.30, on a 1–13 point scale where a score of 13 would indicate maximum distress; minimum score 6 and maximum score 11.55.

### Imaging preprocessing

MRI scanning was conducted at the Medical Research Council Cognition and Brain Sciences Unit on a 3-Tesla Trio Tim MRI scanner by using a head coil gradient set. Whole-brain data were acquired with echoplanar T2* weighted imaging (EPI), sensitive to BOLD signal contrast (48 sagittal slices, 3 mm-thickness; TR = 2400 ms; TE = 30 ms; flip angle = 78°; FOV 192 mm. To provide for equilibration effects, the first 7 volumes were discarded. T1 weighted structural images were acquired at a resolution of 1 × 1 × 1 mm. Statistical parametric mapping software (SPM5) was used to analyze all data. Preprocessing of fMRI data included spatial realignment, coregistration, normalization and smoothing. To control for motion, all functional volumes were realigned to the mean volume. Images were spatially normalized to standard space using the Montreal Neurological Institute (MNI) template with a voxel size of 3 × 3 × 3 mm and smoothed using a Gaussian kernel with an isotropic full width at half maximum (FWHM) of 8 mm. Additionally, high-pass temporal filtering with a cutoff of 128 s was applied to remove low frequency drifts in signal.

### Imaging statistical analysis

After preprocessing, statistical analysis was performed using the general linear model (GLM). Analysis was carried out to establish each participant's voxel-wise activation during the Decide event and Video event (the outcome of the decision: watching the shocks be administered). Activated voxels were identified using an event-related statistical model representing the experimental events, convolved with a canonical hemodynamic response function and mean-corrected. Six head-motion parameters defined by the realignment and were added to the model as regressors of no interest. Contrast images were calculated using GLMs and separately entered into full factorial ANOVAs.

For group statistics, analyses of variance (ANOVAs) were used. We compare the Video event in the PvG task to the Video event in the Non-Moral control task to assess global and small volume corrected activity while watching the decision's outcome. We then used this contrast with covariates of interest — both for the Empathic Concern and Personal Distress subscales of the Interpersonal Reactivity Index (Davis, 1983). A parametric regression analysis was employed to explore which brain regions showed an association with the amount of shock delivered/Money Kept across the PvG task: we use a 1–6 parametric regressors weighted to the money chosen per trial — corresponding to the Likert scale used during the Decide event, coupled with the outcome (Video event). We also use individual scores from the Empathic Concern subscale as a covariate of interest in a second level analysis for these parametric regressions. In addition, we parametrically weight self-reported distress from the previous trial to the current Decision event, in order to explore the effect of situational distress on subsequent choice. Finally, we compute the difference between situational distress reported on the previous trial with the level of shock/money given up on the subsequent trial. This difference score enables explicit indexing of how distress changes choice. We report activity at p < 0.001 uncorrected for multiple spatial comparisons across the whole-brain, and small volume corrected at p < 0.05 FWE on a priori regions of interest; these coordinates were taken from previous related studies associated with empathy, reward and prosocial behavior, and which are proposed by the caregiving model see tables) (Preston, 2013). Altogether, we tested nine different regions of interest that are outlined in the caregiving model to either inhibit or facilitate helping behavior. In addition, we used AlphaSim, an AFNI tool (http://afni.nimh.nih.gov/afni/doc/manual/AlphaSim), to calculate the correct thresholding for multiple comparisons equivalent of p = 0.05 family-wise error rate (FWE). AlphaSim uses the actual data structure to determine the number of independent statistical tests, thus balancing Types I and II errors. With 10,000 Monte Carlo simulations and a voxel-wise significance of p < 0.001, a smoothing kernel of 8-mm FWHM, an overall p = 0.05 FWE corresponded to a cluster extent minimum of 27 voxels for the whole brain.

## Results

### Behavioral results

In order to verify that the PvG task was a sensitive individual differences measure, we first examined how much money subjects retained: on average, subjects kept £12.71, SD ± 4.2 (out of a possible £20, where £20 indicates maximally selfish decisions), with a minimum–maximum spread of £3.80–£19.00. To ensure that altruistic decisions could not be explained by subjects modifying their decisions in response to reputation management or feelings of being watched (Hawthorne effect) (Landsberger, 1958), we examined the correlation between subjects' ratings (8-point VAS) of beliefs about being watched with the amount of Money Kept (r = − .05, p = .84, all correlations are Pearson), providing no support for the Hawthorne effect.

We next verified that subjects exhibited situational distress after watching the outcome of their decisions unfold. This measure (self-reported distress ratings following the Video event) revealed that subjects reported being distressed at watching another in pain (mean 8.2 SD ± 1.3). We should also note that only one subject selected the option ‘no shock’ more than once; but even this subject choose to forgo the money and administer ‘no shock’ on only six of the 20 trials.

**Fig. 2:**
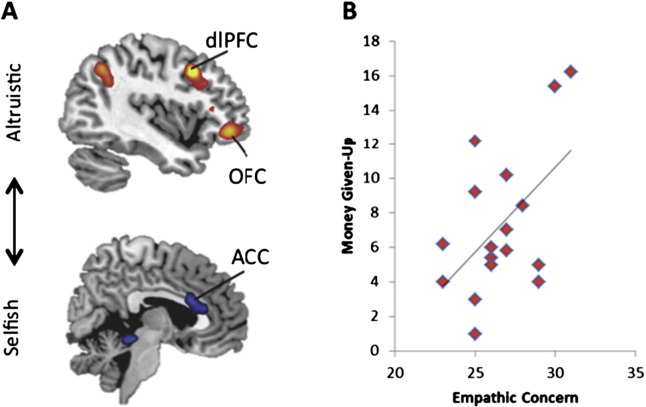
Altruistic action and empathic concern. A. a parametric regression analysis (parametric weights on a scale of 1–6 corresponding with amounts of Money Given-Up/Money Kept made during the decision phase) for the video feedback epoch illustrates that increasingly selfish outcomes are indexed by activity in the dACC while increasingly altruistic outcomes are indexed by activity in the orbital frontal cortex (OFC) dorsolateral prefrontal cortex (dlPFC), and posterior temporal parietal junction (pTPJ). B. A significant correlation (Pearson's 2 tailed: r = 0.53, p = .02) supports the idea that Empathic Concern is associated with costly altruism.

We next investigated whether the Personal Distress and Empathic Concern subscales of the IRI (Davis, 1983) differentially influenced selfish/altruistic choices in the PvG task. In line with the empathy–altruism hypothesis, subjects' trait Personal Distress scores were not significantly correlated with Money Given-Up and the effect size estimate was small (r = .16, p = .53), while trait Empathic Concern did positively correlate with Money Given-Up with a large effect size (r = 0.53, p = .02; [Fig. 2B]). This latter result remained significant even when we controlled for dispositional Personal Distress scores (r_ρ_: r = 0.53, p = .03), indicating that the relationship between trait Empathic Concern and altruistic decisions does not appear to be due to subjects' trait Personal Distress.

Although we found no relationship between trait Personal Distress (Davis, 1983) and costly altruism, we explored whether trial-by-trial situational distress influenced Money Given-up, and whether this might have an interactive effect with trait Empathic Concern. In other words, it is plausible that that the degree of situational distress a subject feels may be a function of an individual's trait levels of Empathic Concern, and together this relationship might have an interactive effect on costly altruism. To test this we conducted repeated measures regressions where level of shock chosen was the dependent variable, situational (trial-by-trial) distress was a lagged predictor (that is, distress on trial 1 predicts choice shock on trial 2), and trait Empathic Concern was the covariate. To test the interactive effect we added the product term of situational distress and trait Empathic Concern as a predictor. We found significant effects of situational distress (β = − .26, SE = .06, p < 0.001) and trait Empathic Concern (β = − .48, SE = .24, p < 0.05) on altruistic giving, but the interactive effect between situational distress and Empathic Concern was not significant (β = .007 SE = .04, p = .86).

Although we found no significant correlation between any of the post scan ratings of responsibility, guilt, and likability/feelings towards the Receiver and Money Given-up, we did find that Empathic Concern (but not Personal Distress or situational distress) positively correlated with likability ratings (r = .50 p = .043) and feelings towards the Receiver (r = .57 p = .017). Furthermore, likability ratings and feelings towards the Receiver were highly correlated with one another (r = .90, p < 0.001).

### Imaging results

Our next goal was to explore whether the feedback from the decision – where subjects were required to watch another in pain – activated the prototypical ‘empathy for pain’ brain regions (Singer et al., 2004). Analyses of the Video event revealed robust bilateral AI and dACC activation at both small volume-corrected and global thresholds [Fig. 1B, Table 1], supporting the well-documented evidence that these regions engage when observing another in pain (Singer et al., 2004; Gu et al., 2010; Fan et al., 2011) as well as during physical pain (Eisenberger and Lieberman, 2004; Atlas et al., 2014). Bilateral right temporoparietal junction (TPJ) – a region linked to theory of mind and emotional perspective-taking (Young et al., 2007) – was also activated, which reinforces the idea that observing another in pain as a consequence of a motivated choice activates regions of the mentalizing network (Zaki and Ochsner, 2012).

#### Modulating the empathy for pain network: the differential neural signatures of empathic concern, personal distress and situational distress

Our next aim was to examine if this empathy for pain network differentially activates while viewing the Video event as a function of Empathic Concern or Personal Distress. First, the fact that we found no intercorrelation between Personal Distress and Empathic Concern led us to hypothesize that these seemingly separable empathy constructs may be differentially represented in the brain. Increasing trait Empathic Concern (subjects' individual IRI Empathic Concern scores entered as a second level covariate for the Video event [contrasted to the control Video event, see Pain vs. Gain (PvG) task section]) revealed greater activation in the anterior temporal lobe (aTL) (Table 2) — a region known to be damaged in those exhibiting inappropriate social behaviors (Bozeat et al., 2000), including loss of insight and reduced empathy for others; these neural activation patterns were also found once we statistically controlled for Personal Distress scores (Table 3). Although we found no behavioral evidence that trait Personal Distress correlated with costly altruism, the equivalent analysis with Personal Distress scores entered as a second level covariate revealed increasing activity in the ACC (Table 4).

Since we also observed that situational distress significantly influenced choice behavior (Behavioral results), we ran this analysis at the neural level. Situational distress (trial-by-trial) was added as a parametric regressor during the Video event. We found robust activation within the OFC (bilaterally) and right TPJ (Table 5), reflecting a possible associative learning signal between situational distress and the outcome of one's moral (or immoral) choice (Kringelbach, 2005; Schoenbaum et al., 2011).

#### Choice behavior and empathy

In order to decompose the relationship between moral choice (i.e. deciding to keep the money and apply electric shocks) and Empathic Concern and Personal Distress, we analyzed the Decide event (parametrically weighted to Money Kept/Given-Up for each subject) with individual Empathic Concern and Personal Distress scores added in as covariates of interest at the second level. The relationship between moral choice and increasing Empathic Concern was indexed by greater activity in the right DLPFC (Table 6).

#### Choice behavior and distress

In contrast, the same analysis with Personal Distress added as a second level covariate of interest revealed increased BOLD activity in the right amygdala and midbrain (Table 7). Next we investigated the Decide event parametrically weighted to situational distress (trial-by-trial), where self-reported distress on the previous trial was tagged to the current Decide event. Increased BOLD activity in the ventral lateral PFC and somatosensory motor area (SMA) indexed the relationship between situational distress and choice (Table 8). However, in order to model the explicit influence of situational, transitory distress on costly altruism, we computed the difference between reported situational distress on the previous trial with shock level/Money Kept on the subsequent trial. That is, we took the difference between distress ratings on trial 1 and the shock level selected on trial 2 and parametrically weighted these difference scores with the Decide event. Whereas higher, positive numbers indicate that increased situational distress boosted altruistic helping, lower, negative numbers indicate that distress had little, or no effect on costly altruism. The relationship between increasing situational distress and helping behavior was indexed by bilateral dlPFC activity (Table 9).

#### Observing the consequences of one's actions

We next investigated the neural circuitry underlying the relationship between an action and its value outcome (action–outcome associations). To directly examine the brain regions associated with watching the consequences of increasingly self-interested versus prosocial behavior, we analyzed the Video event (parametrically weighted to Money Kept/Given-Up during the decision phase for all trials). Greater amounts of Money Kept during the outcome phase (and thus exposure to more aversive feedback) was underpinned by greater activity in the dACC at both global and small volume-corrected thresholds (Fig. 2A, Table 10). This is an area commonly associated with processing aversive (Liu et al., 2007) and emotionally conflicting stimuli (Baumgartner et al., 2009), and which is predicted by the caregiving model to arbitrate between executive control and the instinctual responses of tending to another's needs (Preston, 2013). On the other hand, observing the consequences of progressively prosocial decisions (larger amounts of Money Given-Up) was associated with greater activity in OFC and the posterior portion of the dlPFC (on the border between BA 9 and 44) (Fig. 2A, Table 11) — regions known to support reward valuation (O'Doherty et al., 2001), and which are necessary for top-down processing (Ochsner and Gross, 2005; Preston, 2013).

#### The neural signature of the empathic concern–altruism relationship

**Fig. 3:**
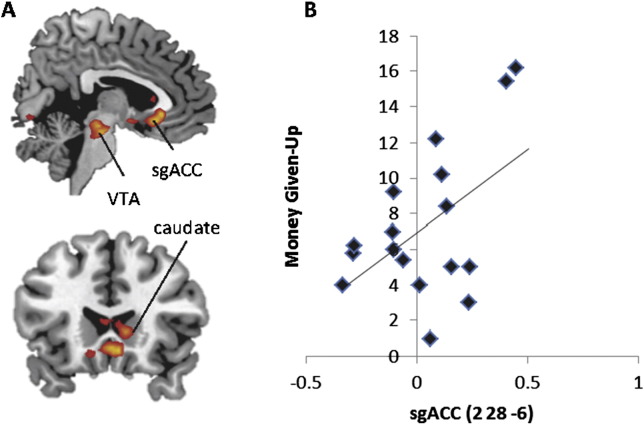
The relationship between altruism and other-oriented empathic concern: A. increasing trait Empathic Concern and motivated choice (parametrically weighted to money given-up) correlates with activation in the VTA, subgenual ACC and caudate. B. sgACC beta values illustrates this correlation between increasing trait empathic concern and costly altruism (z = 3.27, p < .001).

Finally, we explored the action–outcome associations for whether individual trait Empathic Concern was differentially expressed in the brain for such motivated selfish and prosocial behavior. While subjects viewed the Video event, activity in the ventral tegmental area (VTA), subgenual ACC (sgACC) extending to the NAcc, and caudate (all assessed at both global and small volume-corrected thresholds (Fig. 3A, Table 12)) showed a greater association with Money Given-Up in individuals with higher Empathic Concern scores (Money Given-up was entered as parametric regressor at the first level and Empathic Concern scores were entered as a second level covariate). To take account of any effect of individual differences in trait Personal Distress on this empathic concern–altruism relationship – and to mirror the behavioral analysis – we ran this same analysis while covarying Personal Distress. Further confirming the role of this network in supporting other-oriented prosocial decisions, we found that the same regions of the VTA, sgACC, and caudate indexed this empathic concern–altruism relationship (Table 13).

While it is certainly possible that observing the outcome of one's own moral decision may generate feelings of guilt and responsibility – which could account for the activation within the VTA, sgACC and caudate (Drevets and Savitz, 2008) – we found no behavioral evidence that subjects' feelings of guilt (r = − .25, p = .33) or level of responsibility (r = − .12, p = .63) correlated with Money Given-Up. Interestingly however, while there was no significant neural activity associated with increasing guilt, we did observe that decreasing guilt (added as a covariate of interest to the Decide event parametrically weighted to Money kept) was associated with an increased BOLD signal in the sgACC (Table 14), the same region found to index the relationship between increasing Empathic Concern and costly altruism (a conjunction analysis between increasing Empathic Concern and decreasing guilt (Table 15).

## Discussion

A pivotal question that has long concerned philosophers and evolutionary biologists is why do we help strangers in need at a cost to ourselves? In social psychology, two positions have emerged; one proposes that altruistic behavior arises from the desire to reduce our own ‘egoistic’ personal distress when seeing others in need, while another suggests that experiencing feelings of other-oriented empathic concern better predicts altruistic action. In line with the caregiving model, our findings provide evidence that individuals' readiness to help others is driven more by their trait levels of other-oriented empathic concern than by their trait levels of personal distress.

Why would the general tendency towards concern for another motivate altruistic behavior more than the tendency to reduce one's own feelings of distress? In line with the empathy–altruism hypothesis it is possible that while trait empathic concern generates the general tendency towards other-oriented feelings, trait personal distress causes individuals to become more self-focused, biasing downstream behavior (Batson et al., 1987; Preston, 2002). It is also possible that the empathic concern–altruism relationship stems from a basic, evolutionary drive (Preston, 2013) that links other-directed emotions (i.e. the observer's ability to resonate with the target's distressed state) with the positive affect that ensues from caring for another (Preston and de Waal, 2011). Interestingly, the fact that we additionally found that transitory feelings of distress (trial-by-trial) predicted greater altruistic responding suggests that the motivational antecedent of costly altruism probably depends on a complex interplay of more than one emotional state.

In parallel, we found evidence of distinct brain activity associated with viewing the range of consequences of one's choices (i.e. whether they were selfish or altruistic), indicating that differential neurocognitive mechanisms modulate our responses to motivated altruistic behavior. One prediction of the caregiving model is that prefrontal regions typically engage during deliberative choice, such as when conflicting and complex signals require more strategic responding (Preston, 2013). In line with this, we found greater activation in the dlPFC and OFC when watching the outcomes of increasingly altruistic decisions in the PvG task. We also found that the dlPFC indexed the relationship between feeling increasing situational distress after watching the administration of a shock and the subsequent decision to respond more altruistically. That the dlPFC is engaged across a remarkably wide range of tasks, including prosocial (Knoch et al., 2006; Baumgartner et al., 2012) and anti-social decision-making (Glenn et al., 2009; FeldmanHall et al., 2012a), fits with the theory that the dlPFC is necessary for deliberative top-down integration of contextual cues containing competing information. In this case, it is possible that the dlPFC is implementing the decision to forgo money and help another, overriding the putatively instinctual response to benefit financially. This type of response – where the priority is to preserve the welfare of another at the expense of making money – may require greater strategic processing (Miller and Cohen, 2001), a capacity that demands the engagement of more cortical modulatory systems (Preston, 2013).

On the other hand, observing the consequences of one's own selfish behavior was underpinned by the dACC — a region associated with social and physical pain (Eisenberger and Lieberman, 2004) and conflict monitoring between intentions and behavior (Amodio and Frith, 2006). This accords with the notion that the dACC encodes a mismatch between the situation and response (Preston, 2013), such as the emotional conflict engendered by observing the consequences of one's choice to make money by applying painful shocks to another (Etkin et al., 2011). However, considering that increasingly selfish choices are intimately tied to observing the Receiver in increasingly greater pain, it is also possible that the dACC is indexing the observation of this pain increase (Singer et al., 2004).

Increasing trait Personal Distress and decisions to keep the money and apply electric shocks corresponded to heightened BOLD activity in the right amygdala and midbrain. This pattern of results fits with the care-giving model which stipulates that an avoidant, withdrawal response is routed first through the amygdala and then through the midbrain and Periaqueductal Gray (PAG) (Preston, 2013). The amygdala is considered to subserve vigilant responding to novel and threatening stimuli, which along with activation of the midbrain, can produce downstream avoidance responses (Preston, 2013). Distress has long been theorized to produce a withdrawal, avoidant response rather than a helping altruistic one (Batson et al., 1987).

That trait Empathic Concern best predicted costly altruism fits with the broader caregiving theory that harnessing positive, social bonding, and attachment impulses is central in guiding successful altruistic behavior (Preston et al., 2012). An examination of BOLD activation for the relationship between motivated prosocial behavior and trait empathic concern revealed neural evidence in support of this. We found increased BOLD-signal in regions implicated in positive emotional experiences like social reward and attachment (de Quervain et al., 2004; Skuse and Gallagher, 2009): even when controlling for distress, the VTA, caudate, NAcc and sgACC were more active in highly empathically concerned individuals when watching the consequences of their costly altruistic decisions. These regions comprise a core network thought to facilitate helping behavior (Preston and de Waal, 2011), confirming the caregiving model (Preston, 2013) and the prediction that regions explicitly linked to reward seeking behavior (Mirenowicz and Schultz, 1996; King-Casas et al., 2005; Krueger et al., 2007; O'Doherty, 2007) and empathic moral judgments (Zahn et al., 2009) are associated with the action–outcome relationship between costly altruism and an individual's tendency to express feelings of sympathy and compassion for another.

That we found that the sgACC, caudate and VTA indexed the empathy–altruism relationship fits within the broader literature that characterize these brain areas as playing key roles in motivating helping behavior. For example, the sgACC is densely connected with mesolimbic pathways that facilitate the release of oxytocin (Skuse and Gallagher, 2009) – a neuropeptide which bolsters interpersonal trust and cooperation (Zak et al., 2004) – and also sends direct projections to subcortical areas that control autonomic responses (Freedman and Cassell, 1994). The neuroimaging literature indicates that the sgACC engages during charitable donations (Moll et al., 2006) and social bonding (Moll et al., 2007), and clinical work further illustrates that lesions to this area result in blunted responses to emotionally meaningful stimuli (Damasio, 1996). Similarly, the caudate is known to engage during behaviors which are driven by external or internal experiences of reward (Harbaugh et al., 2007), (King-Casas et al., 2005; Fliessbach et al., 2007; Mobbs et al., 2009). Likewise, the VTA processes rewarding stimuli, and recent research have found that the VTA engages during compassion training when subjects respond to another's suffering (Klimecki et al., 2012).

Collectively, evidence of activation within this suite of brain regions supports the proposal that these neural pathways provide an interface between motivational states and behavioral action (Packard and Knowlton, 2002). In other words, the caudate, VTA and sgACC appear to be regulating empathically biased goal-directed behavior (Luo et al., 2011), serving to motivate the subject to respond to the distress of another (Preston, 2013), even if it is at a cost to the self. In fact, these regions have been explicitly associated with integrating reward inputs from the NAcc to stimulate a response that can promote helping and alleviate another's distress (Preston, 2013). Such evidence, along with the result that trait Personal Distress did not predict costly altruism and was indexed by a network (i.e. amygdala, PAG) known to produce avoidant, withdrawal responses, suggests that costly altruism is primarily motivated by Empathic Concern.

Together our findings indicate that subcortical–paralimbic signaling serves a core function to enable successful other-oriented concern, and in turn, prosocial behavior. One speculation is that individuals high in empathic concern make more altruistic choices because they have greater activation in regions key for signaling the motivational urge to respond. Extensive research has examined the dynamic interplay between empathy and social behavior, and yet the question of what motivates costly altruistic action has remained elusive. Here, we illustrate that other-oriented empathic concern is a likely candidate for the proximate mechanism motivating costly altruism, and that individual differences in empathic concern-related brain responses predict such prosocial choice. This data clarifies working models of empathy and social cooperation, and aids in our understanding of how humans interact, connect, and relate with one another.

## Figures and Tables

**Table 1:** We used independent a priori coordinates and small volume corrected at p < 0.05 Family Wise Error (FWE). Coordinates were taken from the original papers cited in the caregiving model (Preston and Hofelich, 2012; Preston, 2013). Peak voxels are presented in the tables at p < 0.001 uncorrected with a minimum cluster threshold of (k = 27), and all images are shown at p < 0.005 uncorrected. Contrast moral video epoch > non-moral video epoch.

Region	Peak MNI coordinates			Z-value
Left AI	− 34	26	2	4.21
Left AI	− 30	20	6	3.40
Right AI	56	24	0	3.86
Right AI	44	32	− 4	4.04
Right TPJ	52	− 40	4	3.28
dACC	− 10	18	30	3.26
Brain stem	0	− 6	0	3.10

A priori regions	MNI coordinates			t-statistic

Left AI	− 30	9	6	5.01
Left AI	− 48	12	− 3	4.26
Right AI	60	15	3	5.30
Right AI	42	27	− 6	5.70

Regions small volume corrected (SVC) at p < 0.05 FWE with a 10 mm sphere using a priori independent coordinates: Singer et al., 2004.

**Table 2:** Contrast moral video epoch > non-moral video epoch with increasing Empathic Concern [IRI] as a covariate.

Region	Peak MNI coordinates			Z-value
Left aTL	− 62	− 8	− 20	3.75
Left hippocampus	− 26	− 28	− 18	3.11
Right hippocampus	24	− 16	− 18	3.67

**Table 3:** Contrast moral video epoch > non-moral video epoch with increasing Empathic Concern as a covariate, controlling for distress.

Region	Peak MNI coordinates			Z-value
Left aTL	− 62	− 8	− 20	3.63
Right hippocampus	24	− 16	− 16	3.70
Left hippocampus	− 28	− 28	− 20	3.41

**Table 4:** Contrast moral video epoch > non-moral video epoch with increasing Personal Distress [IRI] as a covariate.

Region	MNI coordinates			Z-value
ACC	0	30	4	3.97
ACC	18	44	8	3.12

**Table 5:** Video event parametrically weighted to situational distress.

Region	Peak MNI coordinates			Z-value
Left OFC	− 30	56	− 4	4.16
Right OFC	44	54	0	4.12
Precentral	− 40	12	40	3.76
ACC	12	32	32	3.05
rTPJ	50	− 48	38	3.20
Visual cortex	18	− 76	− 6	3.61

**Table 6:** Decide event parametrically weighted to Money Kept/Given-Up with increasing empathic concern added as covariate of interest.

Region	Peak MNI coordinates			Z-value
Right dlPFC	26	40	46	3.72

**Table 7:** Decide event parametrically weighted to Money Kept/Given-Up with increasing personal distress added as covariate of interest.

Region	Peak MNI coordinates			Z-value
Mid brain	8	− 14	− 18	3.37
R angular gyrus	32	− 48	20	4.29
L angular gyrus	− 28	− 50	22	3.64
Amygdala	26	− 2	− 28	3.16

**Table 8:** Decide event parametrically weighted to situational distress on previous trial.

Region	Peak MNI coordinates			Z-value
vlPFC	− 24	60	18	3.20
SMA	16	18	− 58	3.24
Cerebellum	20	− 50	− 50	3.53

**Table 9:** Decide event parametrically weighted to difference between situational distress on previous trial and shock selected on current trial.

Region	Peak MNI coordinates			Z-value
Left precentral gyrus	− 40	− 16	62	4.24
Right precentral gyrus	54	− 26	42	3.97
Left dlPFC	− 24	18	38	3.15
Right dlPFC	28	30	46	3.33
Right mid TPJ	48	− 64	6	3.57

**Table 10:** Video event parametrically weighted to Money Kept.

Region	Peak MNI coordinates			Z-value
dACC	8	20	24	3.10
Mid temporal lobe	− 44	− 70	10	3.94
Periaqueductal gray	6	− 38	− 12	3.00
Left post central gyrus	− 48	− 8	50	3.64

A priori regions	MNI coordinates			t-statistic

dACC	4	20	24	3.80

Regions small volume corrected (SVC) at p < 0.05 FWE with a 10 mm sphere using a priori independent coordinates. Liu et al., 2007.

**Table 11:** Video event parametrically weighted to Money Given-Up.

Region	Peak MNI coordinates			Z-value
Left dlPFC	− 42	14	40	4.05
Left lOFC	− 50	40	− 6	3.53
Left TPJ	− 42	− 56	42	3.05

**Table 12:** Video event parametrically weighted to Money Kept/Given-Up with increasing empathic concern added as covariate of interest.

Region	Peak MNI coordinates			Z-value
VTA	0	− 24	− 12	3.20
NAcc	6	20	− 10	3.20
Subgenual ACC	2	28	− 6	3.27
Right caudate	10	22	2	3.10
Middle frontal gyrus	− 20	58	− 4	3.10
Left hippocampus	− 30	− 24	− 22	4.02
aTL	54	4	− 16	3.88
Right occipital lobe	46	− 78	18	3.79
Left occipital lobe	− 48	− 82	18	3.95

A priori regions	MNI coordinates			t-statistic

VTA	2	− 20	− 16	3.94
Right caudate	12	24	4	3.77
Subgenual ACC	6	36	− 4	4.10

Regions small volume corrected (SVC) at p < 0.05 FWE with a 10 mm sphere using a priori independent coordinates: Krueger et al., 2007; Zahn et al., 2009; King-Casas et al., 2005. Note: Although an a priori ROI was used to determine the VTA, given the small, tightly clustered nature of the nuclei, it is difficult to fully rule out that some activation may also be within the PAG. Higher resolution imaging should be used to confirm these results.

**Table 13:** Video event parametrically weighted to Money Kept/Given-Up with empathic concern — controlling for personal distress.

Region	Peak MNI coordinates			Z-value
sgACC	2	28	− 6	3.44
Right caudate	10	22	2	3.05
VTA	4	− 22	− 18	3.10
Frontal pole	20	66	14	3.39
aTL	54	4	− 16	3.72
Left hippocampus	− 30	− 24	− 22	3.99
Visual cortex	− 48	− 82	18	3.78

A priori regions	MNI coordinates			t-statistic

Subgenual ACC	6	36	− 4	4.51
Right caudate	12	24	4	3.76

Regions small volume corrected (SVC) at p < 0.05 FWE with a 10 mm sphere using a priori independent coordinates: Zahn et al., 2009; King-Casas et al., 2005.

**Table 14:** Decide event parametrically weighted to Money Kept/Given-Up with decreasing guilt added as covariate of interest.

Region	Peak MNI coordinates			Z-value
sgACC	2	18	− 10	3.80

**Table 15:** Conjunction between video epochs parametrically weighted to Money Given up/Kept and Empathic Concern as covariate + decide epoch parametrically weighted to Money Given up/Kept and decreasing guilt.

Region	Peak MNI coordinates			Z-value
sgACC	6	20	− 10	3.79

## References

[bb0005] Akitsuki Y., Decety J. (2009). Social context and perceived agency affects empathy for pain: an event-related fMRI investigation. NeuroImage.

[bb0010] Amodio D.M., Frith C.D. (2006). Meeting of minds: the medial frontal cortex and social cognition. Nat. Rev. Neurosci..

[bb0015] Andreoni J. (1990). Impure altruism and donations to public goods: a theory of warm-glow giving. Econ. J..

[bb0020] Atlas L.Y., Lindquist M.A., Bolger N., Wager T.D. (2014). Brain mediators of the effects of noxious heat on pain. Pain.

[bb0035] Batson C.D., O'Quin K., Fultz J., Vanderplas M., Isen A.M. (1983). Influence of self-reported distress and empathy on egoistic versus altruistic motivation to help. J. Pers. Soc. Psychol..

[bb0030] Batson C.D., Fultz J., Schoenrade P.A. (1987). Distress and empathy: two qualitatively distinct vicarious emotions with different motivational consequences. J. Pers..

[bb0025] Batson C.D., Dyck J.L., Brandt J.R., Batson J.G., Powell A.L., McMaster M.R., Griffitt C. (1988). Five studies testing two new egoistic alternatives to the empathy–altruism hypothesis. J. Pers. Soc. Psychol..

[bb0040] Baumgartner T., Fischbacher U., Feierabend A., Lutz K., Fehr E. (2009). The neural circuitry of a broken promise. Neuron.

[bb0045] Baumgartner T., Knoch D., Hotz P., Eisenegger C., Fehr E. (2012). Dorsolateral and ventromedial prefrontal cortex orchestrate normative choice. Nat. Neurosci..

[bb0050] Bernhardt B.C., Singer T. (2012). The neural basis of empathy. Annu. Rev. Neurosci..

[bb0055] Bozeat S., Gregory C.A., Ralph M.A., Hodges J.R. (2000). Which neuropsychiatric and behavioural features distinguish frontal and temporal variants of frontotemporal dementia from Alzheimer's disease?. J. Neurol. Neurosurg. Psychiatry.

[bb0065] Cialdini R.B., Schaller M., Houlihan D., Arps K., Fultz J., Beaman A.L. (1987). Empathy-based helping: is it selflessly or selfishly motivated?. J. Pers. Soc. Psychol..

[bb0060] Cialdini R.B., Brown S.L., Lewis B.P., Luce C., Neuberg S.L. (1997). Reinterpreting the empathy–altruism relationship: when one into one equals oneness. J. Pers. Soc. Psychol..

[bb0070] Damasio A.R. (1996). The somatic marker hypothesis and the possible functions of the prefrontal cortex. Philos. Trans. R. Soc. Lond. Ser. B Biol. Sci..

[bb0075] Davis M.H. (1983). Measuring individual differences in empathy: evidence for a multidimensional approach. J. Pers. Soc. Psychol..

[bb0080] de Quervain D.J., Fischbacher U., Treyer V., Schellhammer M., Schnyder U., Buck A., Fehr E. (2004). The neural basis of altruistic punishment. Science.

[bb0085] de Vignemont F., Singer T. (2006). The empathic brain: how, when and why?. Trends Cogn. Sci..

[bb0090] de Waal F.B. (2008). Putting the altruism back into altruism: the evolution of empathy. Annu. Rev. Psychol..

[bb0095] Decety J., Michalska K.J., Akitsuki Y. (2008). Who caused the pain? An fMRI investigation of empathy and intentionality in children. Neuropsychologia.

[bb0100] Decety J., Yang C.Y., Cheng Y. (2010). Physicians down-regulate their pain empathy response: an event-related brain potential study. NeuroImage.

[bb0105] Drevets W.C., Savitz J. (2008). The subgenual anterior cingulate cortex in mood disorders. CNS Spectr..

[bb0110] Eisenberg N. (2000). Emotion, regulation, and moral development. Annu. Rev. Psychol..

[bb0115] Eisenberg N., Fabes R.A., Murphy B., Karbon M., Maszk P., Smith M., Oboyle C., Suh K. (1994). The relations of emotionality and regulation to dispositional and situational empathy-related responding. J. Pers. Soc. Psychol..

[bb0120] Eisenberger N.I., Lieberman M.D. (2004). Why rejection hurts: a common neural alarm system for physical and social pain. Trends Cogn. Sci..

[bb0125] Etkin A., Egner T., Kalisch R. (2011). Emotional processing in anterior cingulate and medial prefrontal cortex. Trends Cogn. Sci..

[bb0130] Fan Y., Duncan N.W., de Greck M., Northoff G. (2011). Is there a core neural network in empathy? An fMRI based quantitative meta-analysis. Neurosci. Biobehav. Rev..

[bb0135] FeldmanHall O., Dalgleish T., Thompson R., Evans D., Schweizer S., Mobbs D. (2012). Differential neural circuitry and self-interest in real vs hypothetical moral decisions. Soc. Cogn. Affect. Neurosci..

[bb0140] FeldmanHall O., Mobbs D., Evans D., Hiscox L., Navardy L., Dalgleish T. (2012). What we say and what we do: the relationship between real and hypothetical moral choices. Cognition.

[bb0145] Fliessbach K., Weber B., Trautner P., Dohmen T., Sunde U., Elger C.E., Falk A. (2007). Social comparison affects reward-related brain activity in the human ventral striatum. Science.

[bb0150] Freedman L.J., Cassell M.D. (1994). Relationship of thalamic basal forebrain projection neurons to the peptidergic innervation of the midline thalamus. J. Comp. Neurol..

[bb0155] Glenn A.L., Raine A., Schug R.A. (2009). The neural correlates of moral decision-making in psychopathy. Mol. Psychiatry.

[bb0160] Gu X., Liu X., Guise K.G., Naidich T.P., Hof P.R., Fan J. (2010). Functional dissociation of the frontoinsular and anterior cingulate cortices in empathy for pain. J. Neurosci..

[bb0165] Harbaugh W.T., Mayr U., Burghart D.R. (2007). Neural responses to taxation and voluntary giving reveal motives for charitable donations. Science.

[bb0175] Hein G., Silani G., Preuschoff K., Batson C.D., Singer T. (2010). Neural responses to ingroup and outgroup members' suffering predict individual differences in costly helping. Neuron.

[bb0170] Hein G., Lamm C., Brodbeck C., Singer T. (2011). Skin conductance response to the pain of others predicts later costly helping. PLoS ONE.

[bb0180] Hoffman M. (2000). Empathy and Moral Development: Implications for Caring and Justice.

[bb0185] King-Casas B., Tomlin D., Anen C., Camerer C.F., Quartz S.R., Montague P.R. (2005). Getting to know you: reputation and trust in a two-person economic exchange. Science.

[bb0190] Klimecki O.M., Leiberg S., Lamm C., Singer T. (2012). Functional neural plasticity and associated changes in positive affect after compassion training. Cereb. Cortex.

[bb0195] Knoch D., Pascual-Leone A., Meyer K., Treyer V., Fehr E. (2006). Diminishing reciprocal fairness by disrupting the right prefrontal cortex. Science.

[bb0200] Kosfeld M., Heinrichs M., Zak P.J., Fischbacher U., Fehr E. (2005). Oxytocin increases trust in humans. Nature.

[bb0205] Kringelbach M.L. (2005). The human orbitofrontal cortex: linking reward to hedonic experience. Nat. Rev. Neurosci..

[bb0210] Krueger F., McCabe K., Moll J., Kriegeskorte N., Zahn R., Strenziok M., Heinecke A., Grafman J. (2007). Neural correlates of trust. Proc. Natl. Acad. Sci. U. S. A..

[bb0215] Lamm C., Batson C.D., Decety J. (2007). The neural substrate of human empathy: effects of perspective-taking and cognitive appraisal. J. Cogn. Neurosci..

[bb0230] Lamm C., Porges E.C., Cacioppo J.T., Decety J. (2008). Perspective taking is associated with specific facial responses during empathy for pain. Brain Res..

[bb0225] Lamm C., Decety J., Singer T. (2011). Meta-analytic evidence for common and distinct neural networks associated with directly experienced pain and empathy for pain. NeuroImage.

[bb0235] Landsberger H. (1958). Hawthorne revisited: management and the worker, its critics, and development in human relations in industry.

[bb0240] Liu X., Powell D.K., Wang H., Gold B.T., Corbly C.R., Joseph J.E. (2007). Functional dissociation in frontal and striatal areas for processing of positive and negative reward information. J. Neurosci..

[bb0245] Luo A.H., Tahsili-Fahadan P., Wise R.A., Lupica C.R., Aston-Jones G. (2011). Linking context with reward: a functional circuit from hippocampal CA3 to ventral tegmental area. Science.

[bb0250] Miller E.K., Cohen J.D. (2001). An integrative theory of prefrontal cortex function. Annu. Rev. Neurosci..

[bb0255] Mirenowicz J., Schultz W. (1996). Preferential activation of midbrain dopamine neurons by appetitive rather than aversive stimuli. Nature.

[bb0260] Mobbs D., Yu R., Meyer M., Passamonti L., Seymour B., Calder A.J., Schweizer S., Frith C.D., Dalgleish T. (2009). A key role for similarity in vicarious reward. Science.

[bb0270] Moll J., Krueger F., Zahn R., Pardini M., de Oliveira-Souza R., Grafman J. (2006). Human fronto-mesolimbic networks guide decisions about charitable donation. Proc. Natl. Acad. Sci. U. S. A..

[bb0265] Moll J., de Oliveira-Souza R., Garrido G.J., Bramati I.E., Caparelli-Daquer E.M., Paiva M.L., Zahn R., Grafman J. (2007). The self as a moral agent: linking the neural bases of social agency and moral sensitivity. Soc. Neurosci..

[bb0275] Numan M. (2006). Hypothalamic neural circuits regulating maternal responsiveness toward infants. Behav. Cogn. Neurosci. Rev..

[bb0290] Ochsner K.N., Gross J.J. (2005). The cognitive control of emotion. Trends Cogn. Sci..

[bb0285] O'Doherty J.P. (2007). Lights, camembert, action! The role of human orbitofrontal cortex in encoding stimuli, rewards, and choices. Ann. N. Y. Acad. Sci..

[bb0280] O'Doherty J., Kringelbach M.L., Rolls E.T., Hornak J., Andrews C. (2001). Abstract reward and punishment representations in the human orbitofrontal cortex. Nat. Neurosci..

[bb0295] Packard M.G., Knowlton B.J. (2002). Learning and memory functions of the basal ganglia. Annu. Rev. Neurosci..

[bb0300] Penner L.A., Dovidio J.F., Piliavin J.A., Schroeder D.A. (2005). Prosocial behavior: multilevel perspectives. Annu. Rev. Psychol..

[bb0315] Preston S.D., de Waal F.B. (2002). Empathy: its ultimate and proximate bases. Behav. Brain Sci..

[bb0305] Preston S.D. (2013). The origins of altruism in offspring care. Psychol. Bull..

[bb0310] Preston S.D., de Waal F., Decety J., Cacioppo J. (2011). Altruism. The Handbook of Social Neuroscience.

[bb0320] Preston S.D., Hofelich A.J. (2012). The many faces of empathy: parsing empathic phenomena through a proximate, dynamic-systems view of representing the other in the self. Emot. Rev..

[bb0325] Schoenbaum G., Takahashi Y., Liu T.L., McDannald M.A. (2011). Does the orbitofrontal cortex signal value?.

[bb0330] Singer T., Frith C. (2005). The painful side of empathy. Nat. Neurosci..

[bb0335] Singer T., Seymour B., O'Doherty J., Kaube H., Dolan R.J., Frith C.D. (2004). Empathy for pain involves the affective but not sensory components of pain. Science.

[bb0340] Singer T., Seymour B., O'Doherty J.P., Stephan K.E., Dolan R.J., Frith C.D. (2006). Empathic neural responses are modulated by the perceived fairness of others. Nature.

[bb0345] Skuse D.H., Gallagher L. (2009). Dopaminergic-neuropeptide interactions in the social brain. Trends Cogn. Sci..

[bb0350] Xu X., Zuo X., Wang X., Han S. (2009). Do you feel my pain? Racial group membership modulates empathic neural responses. J. Neurosci..

[bb0355] Young L., Cushman F., Hauser M., Saxe R. (2007). The neural basis of the interaction between theory of mind and moral judgment. Proc. Natl. Acad. Sci. U. S. A..

[bb0360] Zahn R., de Oliveira-Souza R., Bramati I., Garrido G., Moll J. (2009). Subgenual cingulate activity reflects individual differences in empathic concern. Neurosci. Lett..

[bb0365] Zak P.J., Kurzban R., Matzner W.T. (2004). The neurobiology of trust. Ann. N. Y. Acad. Sci..

[bb0370] Zaki J., Ochsner K. (2012). The neuroscience of empathy: progress, pitfalls and promise. Nat. Neurosci..

